# “In a Perfect World, You Wouldn't Have to Work the System to Get the Things You Need to Survive”: A Pilot Study About Trans Health Care Possibilities

**DOI:** 10.1089/trgh.2018.0049

**Published:** 2019-02-08

**Authors:** Ryan E. Pryor, William Vickroy

**Affiliations:** ^1^ Midwifery Institute, Midwifery and Women's Health Programs, Jefferson (Philadelphia University + Thomas Jefferson University), Philadelphia, Pennsylvania.; ^2^ School of Natural Science, Hampshire College, Amherst, Massachusetts.

**Keywords:** focus group, gender non-binary, transgender, trans health

## Abstract

**Purpose:** aTHeNA (a Trans Health Needs Assessment) is a pilot study exploring the perspectives and experiences of trans people in western Massachusetts on and with health care. This study examines research strategies and best practices to improve trans health care by prioritizing the knowledge and recommendations of trans and gender nonbinary people.

**Methods:** aTHeNA is composed of a focus group of eight trans and gender nonbinary individuals held in western Massachusetts in January of 2016 and qualitative analysis of that focus group to synthesize themes. aTHeNA utilized an interdisciplinary approach. Concepts of respect, care, self-definition, and intersectionality informed study design and analysis.

**Results:** Key participant recommendations include providing medical care that recognizes and values client self-knowledge, elimination of gender markers in insurance billing, and comprehensive health care team education across inpatient and outpatient settings.

**Conclusion:** Focus group participants outlined the limitations of current services and envisioned possibilities for a more ideal system. Further research is needed to incorporate trans perspectives into health literature.

## Introduction

aTHeNA (a Trans Health Needs Assessment) is a pilot transgender (trans) health needs assessment, which engages a group of trans and gender non-binary individuals in western Massachusetts to share their knowledge and experience of existing health care systems and their visions for an ideal health care system. It is well documented that transgender people in the United States and in Massachusetts face discrimination in and outside of health care settings.^1^ Western Massachusetts is predominantly rural and regionally underrepresented in terms of trans health research in the state.^2–5^ The rural and semirural parts of the region are predominantly white (non-Latinx). The largest city in the region, Springfield, MA (population 155,000)^6^ has a large Puerto Rican community and relatively large Dominican, Jamaican, Vietnamese, and African American communities. Trans people often travel substantial distances to seek care in the region; even in urban areas, there are a limited number of providers skilled in trans-affirming care.

While aTHeNA is not a Community-Based Participatory Research^7^ (CBPR) or Participatory Action Research (PAR) study, several CBPR and PAR studies conducted by community organizations that included trans participants were influential in our focus group design.^8–10^ Persist Health Project self-published a report “No lectures or stink eye: the health care needs of people in the sex trade,” that asked participants to identify what an ideal health care provider would be like.^8^ Participants wanted providers to address their overall well-being, including mental health, rather than solely focusing on their perceived sexual health risk factors.^8^

Identifying broad definitions of health care issues was crucial in developing a framework for the focus group. The Welfare Warriors Research Collaborative of Queers for Economic Justice in 2010 published “A Fabulous Attitude: Low income LGBTGNC people surviving and thriving on love, shelter, and knowledge,” which defined healing broadly and identified key health care priorities, including access to benefits such as cash benefits, food stamps, and social security, without experiencing negative treatment or discrimination.^9^ They also identified the use of the focus group as a model to build connections and share strategies for resistance.^9^ A 2009 PAR study by the Young Women's Empowerment Project (YWEP) focused on sharing strategies for resilience in the face of violence from individuals and institutions.^10^ YWEP is a youth led organization in Chicago for girls in the sex trade that includes many transgender women. Participants identified many strategies for healing, including taking baths, a good meal, and aromatherapy, expanding the definition of “health” and “care” beyond one that focuses primarily on health care professionals and institutions.

Additional literature of the lived experience of trans people reviewed included a study on LGBTQ experiences of aging^11^ and health care experiences of members of the house/ball community in Charlotte, North Carolina.^12^

### Black feminist theoretical foundations

Patricia Hill Collins' concept of self-definition is a key theoretical construct guiding our research.^13^ Self-definition provides an antidote to the way in which marginalized individuals or groups are often misrepresented by people who do not share their identities—in this case, trans people in medical settings.^13^ Second, the concept of intersectionality informed our methodology. An intersectional analysis posits that forces such as racism, capitalism, and sexism will impact individuals in differing ways based on their identities and experiences.^14^ The concept originated in the 1977 Combahee River Collective Statement and was later coined and expanded upon by critical race theorist Crenshaw.^15,16^ Through an intersectional lens, it is possible to understand that there is no singular trans experience of health care. Finally, how can trans people imagine a world of ideal health care that does not yet exist? The role of radical imagination in the scholarship of Davis,^17^ Gumbs,^18,19^ and the organization Critical Resistance^20^ informed our efforts to center imagination into the framework of the focus group.

### Respect and care

aTHeNA asked participants to establish their own definitions of care and respect with the intention to establish a strong foundation for clinical and institutional recommendations. Respect is a nearly ubiquitous ethical principle referenced in medical and literature, although it is not frequently defined.^21^ A review of the extensive literature on care and caring is outside the scope of this article.

## Study Methods and Design

Human subject protections were addressed throughout the institutional review process, and protocol was established for maintaining research accountability and participant confidentiality. Institutional Review Board approval was obtained through Hampshire College.

### Recruitment

aTHeNA recruited focus group participants using a purposive sampling approach. Transgender participants were invited to participate utilizing a variety of avenues. Recruitment primarily occurred in Hampden County. In person recruitment occurred at two local queer and/or trans organizations, where we also presented the study and invited feedback. Flyers were placed in the lobbies of several sexual health clinics, as well as across community college campuses. Several care providers from area clinics made study contact cards available to their patients. We posted study information on various local queer and trans social media groups and listservs.

Eligible participants were 18 years of age or older, self-identified as transgender and/or gender nonconforming, and were residents of Hampden, Hampshire, Franklin, or Berkshire County. Participant compensation included a $50 gift card, a 1-day bus pass, and a meal during the focus group discussion. We enrolled 10 participants, with 8 in attendance at the focus group session.

### Positionality of authors

In recruiting for aTHeNA, we established our location within trans communities. R.P. identified himself on the study website as a white, transmasculine, queer Family Nurse Practitioner working in a health center in Springfield, Massachusetts, at the time of the focus group. W.V. described himself as a white, cisgender, queer undergraduate student living and studying at Hampshire College in Amherst, MA and an intern at the same health center.

### Discussion guide

In preparation for the focus group, we met with an experienced facilitator of trans youth groups who identifies as AFAB and genderqueer. The primary authors shared goals for the focus group based on our research, and in collaboration with the facilitator, we created a discussion guide to serve as an outline for focus group discussion. The discussion guide (Table 1) was informed by background research and designed to elicit participants' view of existing health care structures in the region, definitions of respect, experiences of care, and expert views and their expertise and experience of respect and care in the context of an ideal health care structure. It was used by the facilitator as a general guide for conversation and questions.

**Table 1.** Discussion Guide

1. What does respect mean to you, in a few words? 2. Describe a moment in your life where you felt cared for. 3. What does health care look like in the area? 4. Who is involved in health care? 5. What are some trans-specific health care services? 6. What about all these things which need to be improved or expanded? (What is missing here? What should care providers be doing to make trans^a^ patients feel safer? How could access improve?) 7. Out of all the health care improvements, our group came up with today, what is one that stands out as having the ability to change your life for the better?

### Data collection

The focus group was held in January 2016 at a community meeting space in the Springfield Central Library. The facility had only gender segregated public restrooms, thus, we chose a meeting room with a private, single stall bathroom to increase participant comfort and safety. Participants were given several documents, including an informed consent document, a confidentiality agreement, a demographics questionnaire (Table 2), and a local trans health resource guide. The demographics questionnaire solicited clients to write in their own identity categories except for education, age, and annual income to utilize language that best reflected client identity. The trans health resource guide was developed by R.P. and other area health care providers based on knowledge of local referral sources and national organizations providing services by phone or Internet. The facilitator reviewed the informed consent with the group and then presented an adaptation of community discussion guidelines designed by AORTA (Anti-Oppression Resource and Training Alliance) to frame a respectful space for conversation.^22^ With permission from participants, the focus group was audio recorded. Participants were invited to choose their pseudonym for our publication.

**Table 2.** Demographics

Demographics survey	Responses (No. of participants, *N*=7)
Pronouns	He/him/his (2)
	They/them or she/her (1)
	He/him/they (1)
	She/her (2)
	They/them (1)
Gender	Female (2)
	FTM, transman (1)
	Genderqueer (1)
	Genderqueer, gender fluid, non-binary (1)
	Male, transmale (1)
	Queer (1)
Race/ethnicity	Asian (1)
	White/Caucasian (6)
Age, years	18–24 (3)
	25–34 (2)
	45–54 (1)
	55–64 (1)
Level of education	High school diploma/GED (1)
	Associates/2 year degree (1)
	Bachelor's/4 year degree (4)
	Master's degree (1)
Annual income (in U.S. Dollars)	<15,000 (2)
	16–25,000 (1)
	26–35,000 (1)
	36–45,000 (1)
	46–54,000 (1)
	Left blank (1)
County of residence	Hampden County, MA (4)
	Hampshire County, MA (3)
What else would you like us to know/what other identities do you hold?	Adopted, Jewish, college student (1)
	Survivor of medical abuse by a medical professional (1)
	Retired (1)
	Queer (1)
	Mentally ill, chronic pain (1)

### Analysis

Transcription was completed using Transcriva.^23^ Multiple independent reviews of the study recording and transcript by the primary authors were completed to identify central themes and quotations which reflected these themes. We further analyzed the data to deduce how current transgender health recommendations in the literature were or were not reflected in the discussion.

### Member checking

Participant recommendations for health care are summarized in Table 3. All eight participants completed an exit survey immediately following the focus group (Table 4). In August of 2018, the five participants who consented to follow up contact in their exit surveys were sent a short online survey which included our summary of recommendations and option to read an article draft (Table 5). Open ended responses in the survey informed our final summary of recommendations (Table 3).

**Table 3.** Summary of Focus Group Recommendations

**Individual level**
Consistently use name and pronoun(s) indicated by client during clinical care
Listen actively and without judgment to clients
Engage with clients and center their knowledge and wishes
Acknowledge and apologize for mistakes made in communication
Seek literature and training for trans health excellence in all aspects of care provision
Avoid making assumptions about clients' gender, pronouns, or sexuality^a^
Apply awareness and understanding that many trans people's experiences of the health care system have been negative in the past—trust building may be necessary^a^
**Systems level**
Establish dedicated, comprehensive trans-affirming health centers
Provide mandatory, comprehensive trans health training in health professions education programs
Create community vetted referral lists, along with guidance for seeking and navigating care
Implement trans-affirming care practices across all medical specialties and health settings
Broaden transition-related insurance coverage (hair removal, chest compression garments, and so on.)
Integrate multiple name, pronoun, and gender options into all EMR systems
Include all-gender bathroom options in all facilities, including clinics, hospitals, gyms, and so on.
Eliminate use of male/female designations in medical billing
Standardized training and expectation in trans health excellence for all staff and providers
Implement institutional trans trainings that go beyond “trans 101” to provide comprehensive on the job training and address the needs of trans employees as well as trans clients^a^
Further research on gender-affirming hormone therapy and gender-confirmation surgeries and their sequelae^a^

^a^ Recommendation from post-focus group member-checking survey.

EMR, electronic medical record.

**Table 4.** Focus Group Exit Survey Results (*N*=8)

	Disagree	Somewhat disagree	Somewhat agree	Agree
In this focus group, we came up with good definitions for care, respect, and health care.	0	0	1	7
The map of health care services we talked about felt true to me.	0	0	2	6
I gained new ideas about health care.	0	1	2	5
I got to use my imagination.	1	0	3	4
I felt safe and comfortable in this space.	0	0	1	7
I was able to share everything I wanted to.	0	1	4	3

**Table 5.** Member-Checking Survey Results (*N*=4)

	Disagree	Somewhat disagree	Somewhat agree	Agree
The recommendations made by aTHeNA would improve my experience of health care	0	0	0	4
The recommendations made by aTHeNA address my concerns adequately	0	0	2	2
The recommendations made by aTHeNA accurately reflect the focus group discussion	0	0	3	1
The recommendations made during this focus group still feel relevant 2 years later	0	1	1	2
There should be similar studies to aTHeNA in the future	0	0	0	4
I would personally participate in more focus groups like aTHeNA	0	0	1	3
I would encourage others to participate in more focus groups like aTHeNA	0	0	2	2

aTHeNA, a Trans Health Needs Assessment.

## Results

### Demographics

Demographics of our focus group are listed in Table 2. Participants were living in Hampden or neighboring Hampshire County. When asked to list words describing their gender, two participants identified as female; one as FTM/transman; one as male/transmale; one as genderqueer; one as genderqueer, fluid, and nonbinary; and one as queer. Six participants identified as white or Caucasian and one as Asian. Five participants were between the ages of 18 and 34, and two were between the ages of 45 and 64. Five out of seven participants had a bachelors level education or above, which included participants currently in a bachelor's program. Annual income ranged from less than 15K–54K. When asked to share other identities that they held, responses included the following: adopted, Jewish, college student, survivor or medical abuse by a medical professional, retired, queer, mentally ill, and chronic pain.

### Respect and care

Clear, nonjudgmental communication was a topic that arose during discussions of respect and care. Care in a medical context was described as providers “checking in,” apologizing for mistakes, listening, asking what name and pronouns someone wanted to use, and providing concrete support. Participants said that they would feel best cared and respected if providers listen to their needs and respect the legitimacy of their knowledge.

[W]hen I know something's wrong, but they're not listening to what I think it is, [they are] not respecting my ability to know myself, and my ability to know what's right and what's wrong within my own body. Listening is key. (Kevin)

Initiating conversation and sharing information to facilitate client's making informed health care choices were identified as provider responsibilities.

…I identified something I wanted to do. Instead of getting harassed or grief for not having done it, the person explored why hadn't I done it, and how could they support me to be in a place where I can do what I wanna follow through on. (Tony)

Authenticity, engagement, and empathy were identified as important provider qualities.

### Mapping trans health care in western Massachusetts

Participants affirmed that there is a shortage in high-quality mental and primary health care providers for trans clients as well as access to specialists, surgeons, and hair removal specialists. Some expressed concerns about the care they were receiving and the motivations of their care providers.

I think what we have is limited, I'd say. Like we have some surgeons, we have some endocrinologists. I feel like we have a lot of people who do it because they know there's a need in the valley, but they don't do it because they want necessarily to support trans folks. It's more of a paycheck. (Ethan)

Participants expressed frustration with having to play the role of medical expert for themselves when seeking care. One participant explained as follows:

I was only there [an inpatient eating disorder clinic] for like two months, and there are like multiple trans people coming in—and they just like, I don't know, like I would tell them repeatedly that I was trans, and what pronouns I use, and it just couldn't get into their heads that that was related to any part of my care. (Pix)

Lack of provider knowledge, for example, about hormone therapy resulted in negative care experiences and sometimes care denial. Lack of space in electronic medical records (EMRs) to record non-legal names and pronouns, and dichotomous categorization of “male” and “female” categories in medical and insurance company billing criteria were barriers to care. Participants reported having to navigate the health care system that was not inclusive of their needs. One participant summarized as follows:

In a perfect world, you wouldn't have to work the system…in order to get the things you need to survive. (Kevin)

### Envisioning the ideal

Participants wanted providers to receive mandatory comprehensive training in the needs of trans patients, and that this training be incorporated into medical and nursing education curricula. Participants recommended integration of correct name, gender, and pronouns in EMRs and scheduling to ensure safety and comfort. Participants identified products such as chest compression garments, hair removal, and menstrual cups as medically necessary and wanted insurance coverage for these items. They also sought trans affirming locations for exercise with accessible locker rooms.

When asked to prioritize what they saw as the most crucial change for them, responses addressed global health concerns. The need for safety from violence inside and outside health care settings was raised as a central need. Support from family was crucial.

I'm a little older, and my kids and stuff… they are still that way. Just to be accepted, to say “yeah, come on over, we're having Christmas here, you're invited.” That's a big thing. It's not healthcare, but it would make my life a lot better. (Marilyn)

Queer and trans content inclusion in high school health classes was desired. Access to trans-affirming mental health services for both trans people and their families was brought up by two participants.

Yeah, I was just thinking about that too, and how for me it would be really important not just to have therapy that I would feel safe going to, but that I would feel safe sending my family too to work through things and learn. (Taylor)

Participant recommendations for trans health care are summarized in Table 3.

## Discussion

Participants in aTHeNA collectively articulated a vision of a health care system within which they would be welcomed to get excellent consistent care, treated with respect, and offered accessible insurance coverage. Many of our findings echo recent medical and nursing literature documenting the lived experience and health care needs of trans people.^8,24–27^ aTHeNA had several limitations. The discussion guide structure, in application, limited the ability of the focus group to imagine alternate health care structures in the time allotted. Future projects should collect, analyze, and present data back to participants in a timely manner to assure quality feedback. A significant limitation is that our sample was not adequately representative of trans people regionally as well as nationally. Our study was based in Hampden County and did not include participants from the two more rural counties in the region. Although as a pilot study, our intent was not to reach saturation; future focus groups would need to be inclusive of trans people with diverse identities and experiences of race, class, gender identity, primary language(s), nationality, sexual orientation, ability, chronic illness, mental illness, religion, and geographic location to identify the lived experience of the wide matrix of barriers to care, and most importantly, all possible solutions and visions for trans health care in the future. aTHeNA contributes to the literature that explores visions of solutions to trans health disparities, generated by trans and gender non-binary people.

## Abbreviations Used

AFAB: assigned female at birth
aTHeNA: a Trans Health Needs Assessment
CBPR: Community-Based Participatory Research
EMRs: electronic medical records
PAR: Participatory Action Research
YWEP: Young Women's Empowerment Project
